# Optimization of the method of measuring left ventricular end-diastolic diameter in cardiac magnetic resonance as a predictor of left ventricular enlargement

**DOI:** 10.1038/s41598-022-12359-2

**Published:** 2022-05-19

**Authors:** Paweł Gać, Łukasz Waszczuk, Jacek Kurcz, Rafał Poręba

**Affiliations:** 1 Department of General and Interventional Radiology and Neuroradiology, Wroclaw Medical University, Borowska 213, 50-556 Wrocław, Poland; 2 Department of Population Health, Division of Environmental Health and Occupational Medicine, Wroclaw Medical University, Mikulicza-Radeckiego 7, 50-368 Wrocław, Poland; 3 Department of Internal Medicine, Occupational Diseases and Hypertension, Wroclaw Medical University, Borowska 213, 50-556 Wrocław, Poland

**Keywords:** Anatomy, Biomarkers, Cardiology, Diseases, Medical research, Pathogenesis, Signs and symptoms

## Abstract

The objective of the study was to optimize the method of measuring left ventricular end-diastolic diameter (LVEDD) in cardiac magnetic resonance (CMR) as a predictor of left ventricular end-diastolic volume (LVEDV). The study group consisted of 78 patients (age 55.28 ± 17.18) who underwent 1.5 T CMR examination. LVEDD measurements in the short axis, in the long axis in the 2-chamber, 3-chamber and 4-chamber views were made by 2 radiologists. The repeatability of LVEDD measurements was assessed. The sensitivity and specificity of various methods of measuring LVEDD as a predictor of left ventricular enlargement (diagnosed based on LVEDV) were assessed. The correlation coefficients between LVEDD measurements made by researcher A and B were 0.98 for the long axis measurements in the 2-chamber and 3-chamber view, and 0.99 for measurements made in the short axis and in the long axis in the 4-chamber view. The lowest LVEDD measurements variability was recorded for the short axis measurements (RD 0.02, CV 1.38%), and the highest for the long axis measurements in the 3-chamber view (RD 0.04, CV 2.53%). In the male subgroup, the highest accuracy in predicting left ventricular enlargement was characterized by the criterion “LVEDD measured in the long axis in the 2-chamber view > 68.0 mm” (accuracy 94.1%). In the female subgroup, the highest accuracy in predicting left ventricular enlargement was achieved by the criterion “LVEDD measured in the short axis > 63.5 mm” (96.3%). In summary, the measurement made in the short axis should be considered the optimal method to LVEDD measure in CMR, considering the repeatability of measurements and the accuracy of left ventricular enlargement prediction.

## Introduction

Left ventricular enlargement, i.e. increasing the dimensions of the left ventricle, is one of the common organic pathologies of the heart, the aetiology of which is heterogeneous and the pathophysiology of which is complex^1^. Left ventricular enlargement can be physiological (athlete’s heart) or pathological. We can distinguish primary causes (enlargement related directly to the pathology of the left ventricular myocardium) and secondary causes (enlargement developing because of pathology not directly related to the left ventricular myocardium) of left ventricular enlargement^2,3^. The primary causes of left ventricular enlargement may be manifested by left ventricular myocardial hypertrophy (hypertrophic cardiomyopathy) and left ventricular dilatation (dilated cardiomyopathy)^4^. Secondary left ventricular enlargement may occur as a result of increased preload or afterload, i.e., volume or pressure overload. The most common causes of left ventricular enlargement in the pathomechanism of volume overload include mitral and aortic valve insufficiency, ventricular septal defect, and patent ductus arteriosus. The causes of left ventricular enlargement due to pressure overload include arterial hypertension, aortic stenosis and aortic coarctation^1,5^.

The diagnosis of left ventricular enlargement is based on imaging methods, most often echocardiography and magnetic resonance imaging (MRI), although other diagnostic imaging methods may also be useful^6^. Echocardiography is a routine, widely available and standardized method. To diagnose left ventricular enlargement in echocardiography, the criterion of left ventricular end-diastolic diameter (LVEDD) is used measured in M-Mode or directly in 2D in the parasternal long axis view, exceeding 58.4 mm in men and 52.2 mm in women^7^. MRI is the “gold standard” in the assessment of the morphology and function of the heart, mainly due to the clearly better differentiation of the anatomical boundary between the endocardium and the lumen of the left ventricle^8^. In the assessment of cardiac magnetic resonance (CMR), left ventricular enlargement is most often diagnosed based on the left ventricular end-diastolic volume (LVEDV), the normative values of which were determined independently for different gender and age, both for absolute values and values indexed in relation to the body surface area^9^.

Assessment of LVEDV, despite the continuous development of equipment and MRI applications, still requires dedicated technical solutions, manual verification of the semi-automatic estimation and experience of the assessor. Moreover, it is time consuming. While numerous attempts are being made to introduce artificial intelligence algorithms into the functional assessment of cardiac cavities, so far fully automated, precise and completely repeatable algorithms are a goal rather than a widely available clinical practice. Measurement of left ventricular end-diastolic diameter, a parameter routinely used in echocardiography, is relatively simple, not time-consuming and does not require dedicated tools. Even though the standard CMR assessment also includes linear measurements, including LVEDD measurement, the recommendations of scientific societies and research groups lack a standardized method of measuring this parameter. Obviously, no attempt should be made to replace a target LVEDV measurement with a LVEDD measurement. However, because the recipients of CMR results are cardiology clinicians who routinely use the LVEDD parameter in echocardiography, attempts should be made to standardize the LVEDD measurement. These tests should be aimed at minimizing the discrepancy of the LVEDD measurement with the LVEDV measurement. Therefore, it seems cognitively interesting and clinically necessary to verify the repeatability of various methods of LVEDD measuring in CMR, as well as to determine the accuracy of various methods of making this measurement as a predictor of LVEDV.

The objective of the study was to optimize the method of LVEDD measuring in CMR as a predictor of LVEDV.

## Materials and methods

The research has been conducted in compliance with the principles of Good Clinical Practice and Declaration of Helsinki. Ethics Committee of Wroclaw Medical University approved the experimental protocol (protocol code KB-414/2021). The written informed consent has been obtained from all the patients taking part in the research. All data collected from the patients were anonymized.

The retrospective study included 78 consecutive patients who came to the MRI Laboratory of the Wroclaw Medical University for CMR in the period from November 15, 2020 to March 15, 2021. The mean age of the patients was 55.28 ± 17.18 years, the youngest patient was 22 years old, and the oldest was 87 years old. In the study group, 65.4% were men and 34.6% were women. The clinical indications constituting the reason why patients were referred for MRI included: heart failure (42 patients), cardiomyopathy (49) [including hypertrophic cardiomyopathy (16), dilated cardiomyopathy (26), arrhythmogenic right ventricular cardiomyopathy (2), left ventricular noncompaction (5)], myocarditis (9), coronary artery disease (15), arrhythmias (1), cardiac involvement in the course of systemic diseases (3) and pericardial pathologies (1). The characteristics of the study group are presented in Table 1.

**Table 1:** Clinical characteristics of the studied group of patients (n = 78).

	X	Me	Min	Max	SD
Age (years)	55.28	58.00	21.00	86.00	17.18

	n		%		
Male gender	51		65.4		
Female gender	27		34.6		
Arterial hypertension	36		46.1		
Type 2 diabetes	7		8.9		
Heart failure	42		53.8		
Myocardial infarction	15		19.2		

	X	Me	Min	Max	SD
LA (mm)	32.32	30.00	20.00	65.00	10.20
Ao (mm)	32.81	33.00	25.00	45.00	4.69
LVEDD (mm)	58.31	56.00	40.00	75.50	9.72
aIVSDD (mm)	10.68	10.00	6.00	24.00	4.04
PWDD (mm)	7.96	7.00	4.00	20.00	2.49
LVEDV (ml)	148.56	140.00	48.20	317.00	53.92
LVESV (ml)	72.49	52.90	14.80	278.90	48.58
LVEF (%)	55.00	59.00	12.00	76.00	15.39
LVM (g)	116.63	106.00	48.00	295.00	47.80

*aIVSDD* anterior interventricular septum diastolic dimension, *Ao* aortic root dimension, *LA* left atrium dimension, *LVEF* left ventricular ejection fraction, *LVEDD* left ventricular end-diastolic diameter, *LVEDV* left ventricular end-diastolic volume, *LVESV* left ventricular end-systolic volume, *LVM* left ventricular mass, *Max* maximum value, *Me* median, *Min* minimum value, *n* number of patients, *PWDD* posterior wall diastolic dimension, *SD* standard deviation, *X* arithmetic mean.

To consider age and gender to optimize the method of LVEDD measuring in CMR as a predictor of LVEDV, subgroups were distinguished. Based on the gender criterion, a male subgroup (n = 51) and a female subgroup (n = 27) were established, while based on the age median (Me = 58 years), a group of patients aged < median (< 58 years, n = 37) and a group of patients aged ≥ median (≥ 58 years, n = 41) were established.

CMR was performed using one 1.5 T Signa Hdx MRI (General Electric Medical Systems, USA) device. CMR was performed according to a standard protocol. ECG gating and breath hold during the gating were used. The study protocol included locating sequences, low-resolution single-shot black blood lookup sequences (Single Shot FSE), CINE steady-state free precession sequences (SSFP), T2 fat saturation sequences (T2fs), and late gadolinium enhancement sequences (IRPrepFGRE). The study protocol consisted of a bolus injection of gadobutrol (Gadovist, Bayer Healthcare, Germany) in the amount of 0.2 mmol/kg body mass through the ulnar fossa veins.

CMR images obtained during the study were assessed using the GE ReportCARD Package (General Electric Medical Systems, USA), a standard application for CMR post-processing assessment provided by the MRI manufacturer. The standard assessment of CMR included the initial assessment of the mediastinal anatomy in the Single Shot FSE sequences, the assessment of the dimensions and volume of the heart chambers in the CINE sequences recorded in the short axis of the left ventricle and in the long axis in the 2-chamber, 3-chamber and 4-chamber views, and the assessment of morphological changes of the left ventricular myocardium (oedema and fibrosis) in T2fs and LGE sequences. In the standard CMR evaluation, the left atrium (LA) and the aortic root (Ao) dimensions were measured in the 3-chamber view; in the short axis view, the left ventricular end-diastolic diameter (LVEDD), anterior interventricular septum diastolic dimension (aIVSDD) and posterior wall diastolic dimension (PWDD) were measured in the basal-middle layers. Based on CINE images in the short axis of the left ventricle, the functional parameters of the left ventricle were measured using the volumetric method. Outlines of the epicardium and endocardium were made in successive layers of the short axis of the left ventricle in diastole and systole. Left ventricular end-diastolic and end-systolic volumes (LVEDV and LVESV) were calculated by summing the consecutive left ventricular lumen areas from the successive layers multiplied by the thickness of the layers. Left ventricular ejection fraction (LVEF) was calculated according to the mathematical formula: LVEF = (LVEDV − LVESV)/LVEDV × 100%. The application, considering the adopted standard myocardial density, estimated the left ventricular mass (LVM) based on the total volume of the left ventricular myocardium. Standard assessment of CMR was performed each time by one of two radiology and imaging diagnostics specialists with experience in the performance and interpretation of CMR reaching up to 150 examinations interpreted annually over the last 5–10 years.

**Figure 1:**
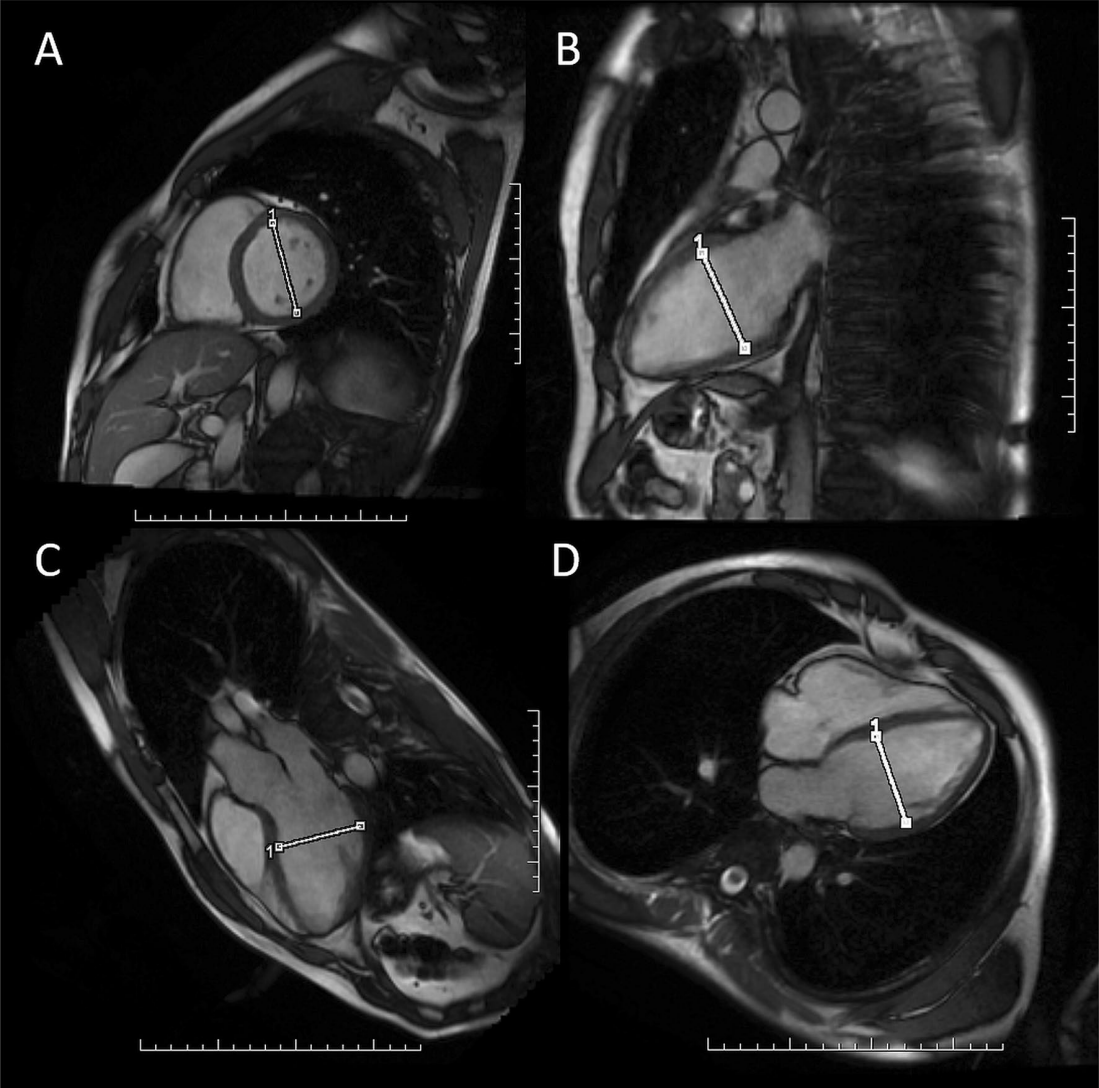
The method of measuring the left ventricular end-diastolic diameter in CMR: (**A**) in the short axis, (**B**) in the long axis in the 2-chamber view, (**C**) in the long axis in the 3-chamber view, (**D**) in the long axis in the 4-chamber view.

The CINE SSFP images obtained during CMR were used to retrospectively perform additional measurements of the left ventricular dimensions in diastole. In addition to LVEDD measured in the basal-middle layers in the short axis view, measurements of LVEDD were made in the long axis in the 2-chamber, 3-chamber and 4-chamber views. LVEDD in the long axis views was measured at 1/3 of the proximal distance of the mitral valve from the apex (Fig. 1). For the purposes of the present study, additional measurements of the left ventricle dimensions were performed independently by two radiologists: one with almost 10 years of professional experience in the evaluation of CMR (researcher A), the other during training in the assessment of CMR (researcher B).

To assess the repeatability of the measurements of the left ventricular dimensions in diastole, secondary parametric variables were calculated, being the resultant of the measurement of LVEDD measured by researcher A and the measurement of the analogous diameter measured by researcher B. The calculated secondary variables and the formulas used to calculate them are as follows: mean measurement (mean, X): X = (measurement A + measurement B)/2; standard deviation of the measurement (standard deviation, SD): SD = √(((measurement A − X measurement)^2^) + ((measurement B − X measurement)^2^))/2); absolute difference (AD): AD =|A measurement − B measurement|; relative difference (RD): RD = AD of measurement/X of measurement; and the coefficient of variation (CV): CV = (SD of measurement/X of measurement) × 100%.

The statistical evaluation of the results was performed with the use of the “Dell Statistica 13” program (Dell Inc., USA). Quantitative variables were characterized by arithmetic means and standard deviations. The results for the qualitative variables were characterized by percentage values. The Shapiro–Wilk W test was used to assess the distribution of variables. In comparative analyses, the test for unrelated variables (in the case of normal distribution of variables) or the Mann–Whitney U test (in the absence of normal distribution of variables) was used to test the hypotheses. To determine the correlations between the studied variables, the Pearson or Spearman correlation analysis and the univariate regression analysis were performed. Moreover, the sensitivity and specificity analysis were performed with the use of ROC (Receiver Operating Characteristic) curve analysis. The results at the levels p < 0.05 were considered statistically significant.

### Ethics approval and consent to participate

All procedures performed in studies involving human participants were in accordance with the ethical standards of the Ethics Committee of Wroclaw Medical University (protocol code KB-414/2021) and with the 1964 Helsinki declaration and its later amendments or comparable ethical standards.

### Consent for participation

Informed consent was obtained from all individual participants included in the study.

## Results

Analysing LVEDD measurements, no statistically significant differences were observed between the mean measurements made by researcher A and researcher B. The correlation coefficients between LVEDD measurements made by researcher A and researcher B were 0.98 for the long axis measurements in the 2-chamber and 3-chamber view, and 0.99 for measurements made in the short axis and in the long axis in the 4-chamber view. The mean values of LVEDD measured in the short axis and in the long axis in the 2-chamber view were about 20% greater than those measured in the long axis in the 3-chamber and 4-chamber view (58.06 mm and 57.42 mm vs. 51.47 mm and 52.52 mm, respectively). The smallest absolute difference in the measurement concerned the measurement made in the short axis (1.10 mm), and the largest absolute difference in the measurement made in the long axis in the 2-chamber view (1.95 mm). In terms of objectified parameters characterizing the repeatability of the measurements, i.e. the relative difference and the coefficient of variation, the lowest values were recorded for the short axis measurements (RD 0.02, CV 1.38%), and the highest for the long axis measurements in the 3-chamber view (RD 0.04, CV 2.53%). The results of analysing the repeatability of LVEDD measurements in CMR are presented in Table 2.

**Table 2:** Repeatability analysis of left ventricular end-diastolic diameter measurements in CMR (n = 78).

	Left ventricular end-diastolic diameter			
	Short axis measurement	Long axis measurement in 2-chamber view	Long axis measurement in 3-chamber view	Long axis measurement in 4-chamber view
Researcher A (mm)	58.26 ± 9.71	57.92 ± 9.87	52.04 ± 9.28	51.86 ± 9.99
Researcher B (mm)	57.87 ± 9.64	56.92 ± 9.74	50.90 ± 9.05	51.18 ± 9.45
p A vs. B	ns	ns	ns	ns
r A vs. B	0.99	0.98	0.98	0.99
X of measurement (mm)	58.06 ± 9.65	57.42 ± 9.75	51.47 ± 9.12	51.52 ± 9.70
SD of measurement (mm)	0.78 ± 0.60	1.38 ± 0.87	1.30 ± 0.74	0.99 ± 0.61
AD of measurement [mm]	1.10 ± 0.85	1.95 ± 1.23	1.83 ± 1.05	1.40 ± 0.86
RD of measurement	0.02 ± 0.02	0.03 ± 0.02	0.04 ± 0.02	0.03 ± 0.02
CV of measurement (%)	1.38 ± 1.10	2.41 ± 1.49	2.53 ± 1.41	1.95 ± 1.19

Values are presented as mean ± standard deviation.

*AD* absolute difference, *CV* coefficient of variation, *ns* non-statistically significant, *p* statistical significance coefficient, *r* correlation coefficient, *RD* relative difference, *SD* standard deviation, *X* arithmetic mean.

Comparing the parameters characterizing the repeatability of LVEDD measurements in CMR, no significant differences were found between the subgroups established based on gender and age median. Gender and age did not affect the repeatability of LVEDD measurements made by researcher A and researcher B. The results of the analysis of the repeatability of LVEDD measurements in CMR in the subgroups established based on gender are presented in Table 3, and in the subgroups created based on the age median in Table 4.

**Table 3:** Repeatability analysis of left ventricular end-diastolic diameter measurements in CMR in gender subgroups.

	Men (n = 51)	Women (n = 27)	p
**Left ventricular end-diastolic diameter**			
**Short axis measurement**			
AD of measurement (mm)	1.20 ± 0.87	0.93 ± 0.78	ns
RD of measurement	0.02 ± 0.02	0.02 ± 0.01	ns
CV of measurement (%)	1.45 ± 1.12	1.24 ± 1.06	ns
**Long axis measurement in 2-chamber view**			
AD of measurement (mm)	1.98 ± 1.22	1.89 ± 1.25	ns
RD of measurement	0.03 ± 0.02	0.03 ± 0.02	ns
CV of measurement (%)	2.39 ± 1.46	2.46 ± 1.58	ns
**Long axis measurement in 3-chamber view**			
AD of measurement (mm)	1.86 ± 1.06	1.78 ± 1.05	ns
RD of measurement	0.04 ± 0.02	0.04 ± 0.02	ns
CV of measurement (%)	2.48 ± 1.33	2.61 ± 1.57	ns
**Long axis measurement in 4-chamber view**			
AD of measurement (mm)	1.47 ± 0.88	1.26 ± 0.81	ns
RD of measurement	0.03 ± 0.02	0.03 ± 0.02	ns
CV of measurement (%)	1.97 ± 1.16	1.91 ± 1.28	ns

Values are presented as mean ± standard deviation;

*AD* absolute difference, *CV* coefficient of variation, *RD* relative difference.

**Table 4:** Repeatability analysis of left ventricular end-diastolic diameter measurements in CMR in age subgroups.

	Age ≥ median (≥ 58 years; n = 41)	Age < median (< 58 years; n = 37)	p
**Left ventricular end-diastolic diameter**			
**Short axis measurement**			
AD of measurement [mm]	1.10 ± 0.80	1.11 ± 0.91	ns
RD of measurement	0.02 ± 0.01	0.02 ± 0.02	ns
CV of measurement (%)	1.34 ± 0.99	1.42 ± 1.22	ns
**Long axis measurement in 2-chamber view**			
AD of measurement [mm]	1.88 ± 1.21	2.03 ± 1.26	ns
RD of measurement	0.03 ± 0.02	0.04 ± 0.02	ns
CV of measurement (%)	2.30 ± 1.49	2.54 ± 1.51	ns
**Long axis measurement in 3-chamber view**			
AD of measurement [mm]	1.93 ± 1.13	1.73 ± 0.96	ns
RD of measurement	0.04 ± 0.02	0.03 ± 0.02	ns
CV of measurement (%)	2.61 ± 1.47	2.43 ± 1.35	ns
**Long axis measurement in 4-chamber view**			
AD of measurement [mm]	1.39 ± 0.86	1.41 ± 0.86	ns
RD of measurement	0.03 ± 0.02	0.03 ± 0.02	ns
CV of measurement (%)	1.84 ± 1.13	2.07 ± 1.27	ns

Values are presented as mean ± standard deviation.

*AD* absolute difference, *CV* coefficient of variation, *RD* relative difference.

Correlation analysis showed the existence of statistically significant positive linear relationship between LVEDD measured using each of the assessed methods and LVEDV. Correlation coefficients, depending on the method of LVEDD measuring and the researcher who made the measurements, ranged from 0.81 to 0.84. The complete results of the correlation analysis in the study group are presented in Table 5.

**Table 5:** Results of the correlation analysis in the studied group of patients (n = 78).

Left ventricular end-diastolic diameter (mm)	Left ventricular end-diastolic volume (ml)
**Short axis measurement**	
Researcher A measurement	0.82
Researcher B measurement	0.81
Mean measurement	0.82
**Long axis measurement in 2-chamber view**	
Researcher A measurement	0.83
Researcher B measurement	0.83
Mean measurement	0.83
**Long axis measurement in 3-chamber view**	
Researcher A measurement	0.81
Researcher B measurement	0.84
Mean measurement	0.83
**Long axis measurement in 4-chamber view**	
Researcher A measurement	0.83
Researcher B measurement	0.83
Mean measurement	0.83

The table shows the correlation coefficient of statistically significant correlations (p < 0.05).

Using the univariate regression analysis, mathematical formulas were created, by means of which, based on the measured left ventricular end-diastolic diameters, the value of the LVEDV can be estimated:

- LVEDV [ml] = 4.561 LVEDD measured in the short axis [mm] − 116.278 (corrected R2: 0.83)
- LVEDV [ml] = 4.612 LVEDD measured in the long axis in the 2-chamber view [mm] − 116.259 (corrected R2: 0.82)
- LVEDV [ml] = 4.898 LVEDD measured in the long axis in the 3-chamber view [mm] − 103.513 (corrected R2: 0.83)
- LVEDV [ml] = 4.626 LVEDD measured in the long axis in the 4-chamber view [mm] − 89.801 (corrected R2: 0.83)

**Figure 2:**
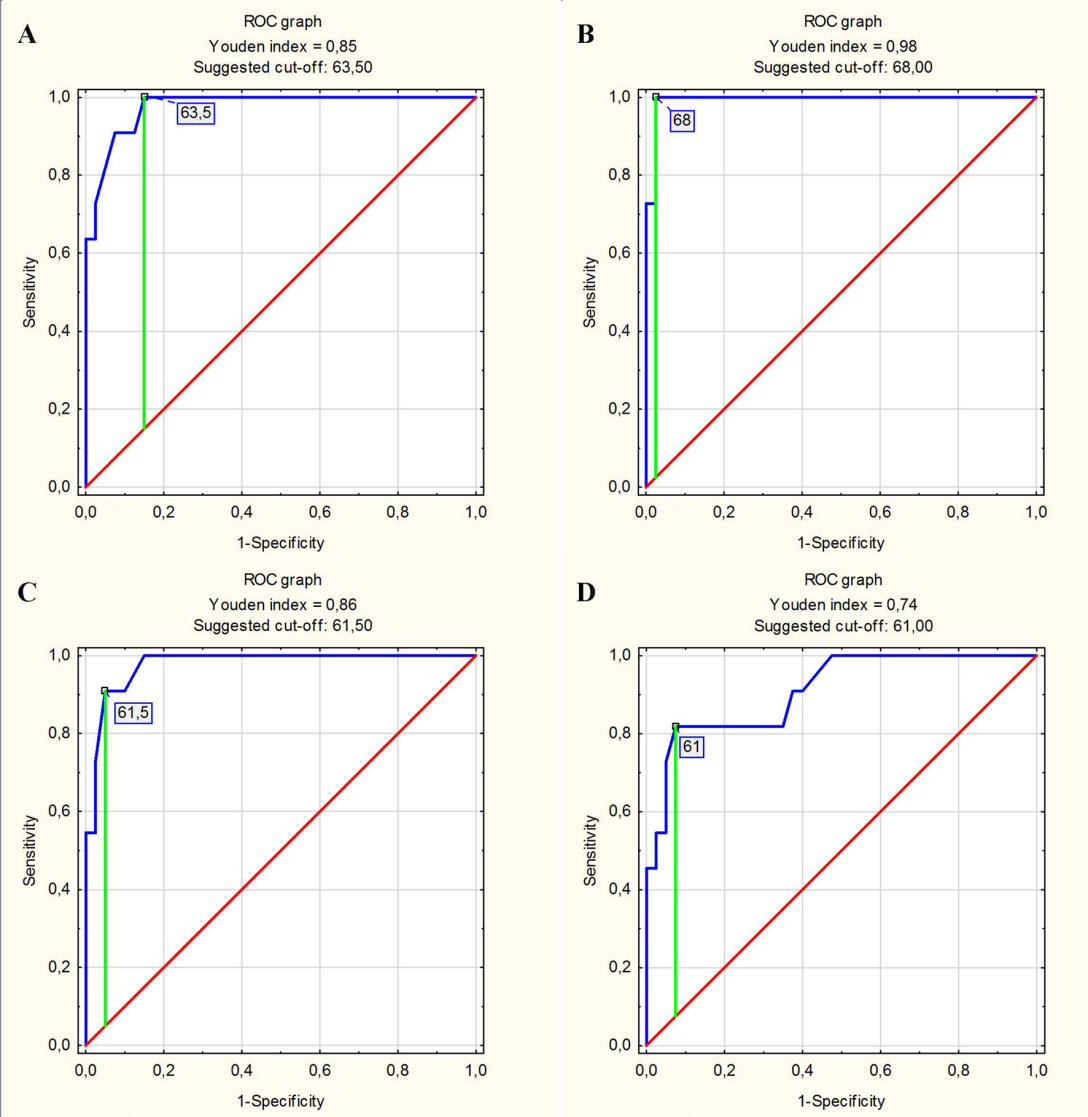
ROC curves determining the optimal cut-off points for left ventricular end-diastolic diameter (LVEDD) measurements in the prediction of left ventricular enlargement diagnosed based on left ventricular end-diastolic volume in the male subgroup: () LVEDD measured in the short axis as a predictor of left ventricular enlargement. (**B**) LVEDD measured in the long axis in the 2-chamber view as a predictor of left ventricular enlargement. (**C**) LVEDD measured in the long axis in the 3-chamber view as a predictor of left ventricular enlargement. (**D**) LVEDD measured in the long axis in the 4-chamber view as a predictor of left ventricular enlargement.

**Figure 3:**
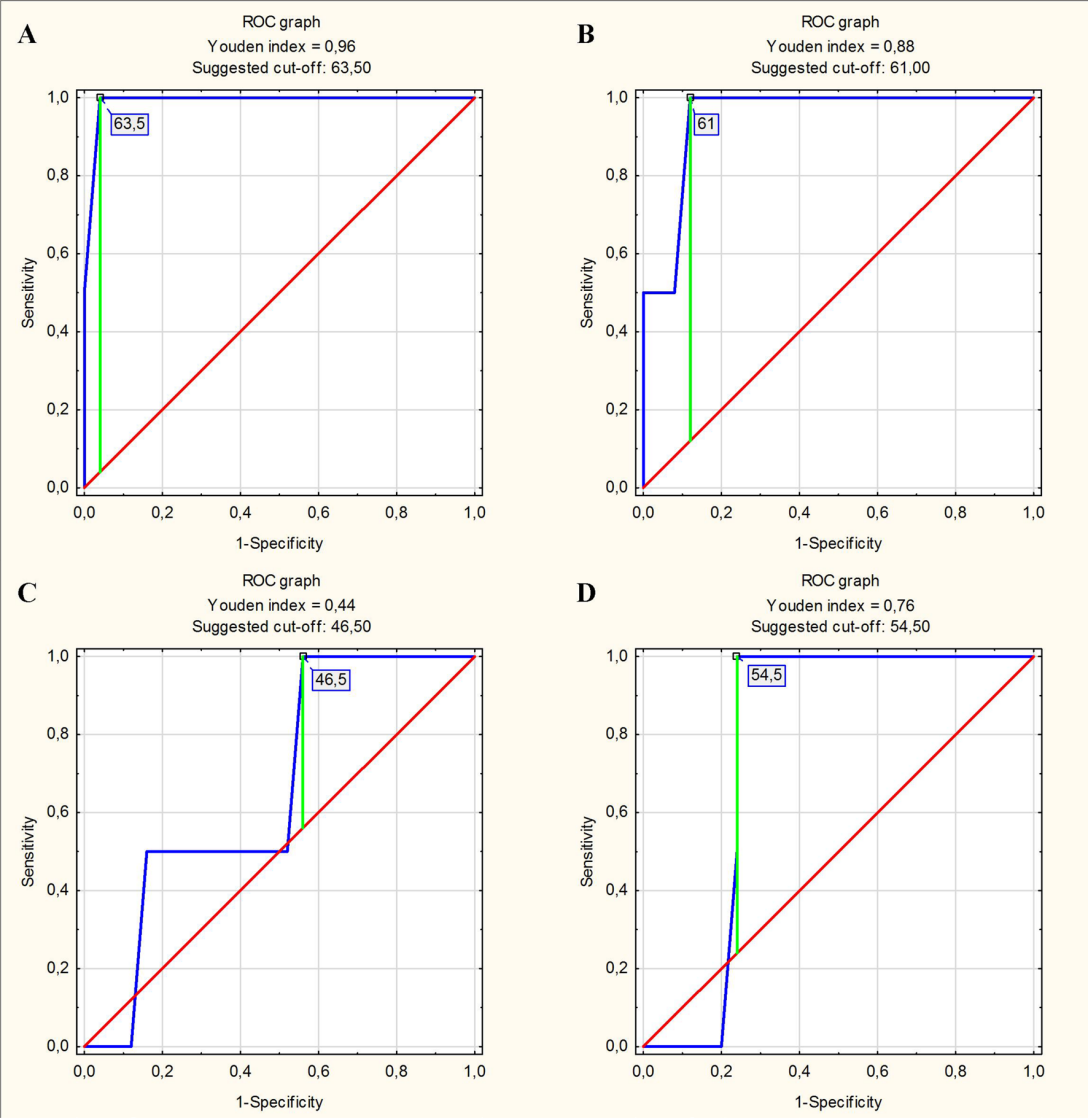
ROC curves determining the optimal cut-off points for left ventricular end-diastolic diameter (LVEDD) measurements in the prediction of left ventricular enlargement diagnosed based on left ventricular end-diastolic volume in the female subgroup: (**A**) LVEDD measured in the short axis as a predictor of left ventricular enlargement. (**B**) LVEDD measured in the long axis in the 2-chamber view as a predictor of left ventricular enlargement. (**C**) LVEDD measured in the long axis in the 3-chamber view as a predictor of left ventricular enlargement. (**D**) LVEDD measured in the long axis in the 4-chamber view as a predictor of left ventricular enlargement.

Using the sensitivity and specificity analysis, the accuracy of LVEDD (assessed by various means) as a predictor of left ventricular enlargement (diagnosed based on LVEDV in CMR) was evaluated. LVEDD measurement values representing the optimal cut-off points for the prediction of left ventricular enlargement in the male subgroup (Fig. 2A–D) and in the female subgroup (Fig. 3A–D) were determined based on successive ROC curves. In the male subgroup, the highest accuracy in predicting left ventricular enlargement was characterized by the criterion “LVEDD measured in the long axis in the 2-chamber view > 68.0 mm”. The accuracy for this criterion was 94.1%. The accuracy of the other predictors ranged from 88.2 to 92.2%. In the female subgroup, the highest accuracy in predicting left ventricular enlargement was achieved by the criterion “LVEDD measured in the short axis > 63.5 mm”. The accuracy of this criterion was 96.3%. For the remaining predictors, the accuracy ranged from 48.1 to 88.9%. Table 6 summarizes the overall results of the sensitivity and specificity analysis on the prediction of left ventricular enlargement based on the LVEDD measured in CMR.

**Table 6:** Prediction of left ventricular enlargement (diagnosed based on left ventricular end-diastolic volume) based on left ventricular end-diastolic diameter measured in CMR.

Real state	Predictor	Predictor evaluation parameters		
		Sensitivity	Specificity	Accuracy
**A. In the male group**				
Left ventricular enlargement (diagnosed based on LVEDV)	LVEDD measured in the short axis > 63.5 mm	0.875	0.909	0.882
	LVEDD measured in the long axis in the 2-chamber view > 68.0 mm	0.975	0.818	0.941
	LVEDD measured in the long axis in the 3-chamber view > 61.5 mm	0.975	0.727	0.922
	LVEDD measured in the long axis in the 4-chamber view > 61.0 mm	0.950	0.727	0.902
**B. In the female group**				
Left ventricular enlargement (diagnosed based on LVEDV)	LVEDD measured in the short axis > 63.5 mm	1.000	0.500	0.963
	LVEDD measured in the long axis in the 2-chamber view > 61.0 mm	0.920	0.500	0.889
	LVEDD measured in the long axis in the 3-chamber view > 46.5 mm	0.480	0.500	0.481
	LVEDD measured in the long axis in the 4-chamber view > 54.5 mm	0.760	0.500	0.741

## Discussion

LVEDD measurements by CMR by 2 different researchers are highly reproducible. The repeatability of LVEDD measurements by 2 researchers is high, regardless of the method used to measure it. Measurements made by different researchers are strongly correlated with each other, they do not differ statistically from each other, and their coefficients of variation are in the range of 1.38–2.53%. Based on the obtained results, it can be indicated, however, that the optimal way to LVEDD measure in CMR (due to its highest repeatability) is the measurement carried out in the short axis. The objectified parameters characterizing the repeatability of the measurements were the lowest. The repeatability of LVEDD measurements by MRI is independent of gender and age. At the same time, it should be remembered that, depending on the method of measuring left ventricular end-diastolic diameter, MRI results in values that differ by up to 20%. Based on the obtained results, it is impossible to clearly define which of the assessed methods of LVEDD measuring by MRI is the optimal method in the context of predicting left ventricular enlargement. The correlation coefficients between LVEDD measured by various methods and LVEDV were similar, and the accuracy of subsequent predictors suggested by the analysis of ROC curves in the studied male subgroup was within a narrow range of 88.2–94.1%. The varied accuracy of successive predictors suggested based on the analysis of ROC curves in the studied female subgroup, ranging from 48.1 to 96.3%, different from that obtained in the male subgroup, has limited reliability. It results from the extremely small subgroup of patients with diagnosed left ventricular enlargement, amounting to 2 cases among the women participating in the study. To sum up, considering the repeatability of measurements and the accuracy of the prediction of left ventricular enlargement, the measurement in the short axis should be considered the most appropriate method to LVEDD measure in MRI. At the same time, it should be noted that measurements in the long axis do not give significantly different results, assuming the use of different cut-off points when diagnosing left ventricular enlargement.

The issue of repeatability of measurements in diagnostic tests is one of the key issues in the context of their clinical usefulness. Despite numerous scientific studies on the repeatability of MRI measurements, the issue of the repeatability of LVEDD measurements has not been discussed so far.

In the existing scientific literature, most attention in the research on the variability of measurements in CMR has been devoted to the issue of estimating the volume of the left and right ventricles and the mass of the left ventricular myocardium. Already in the 1990s, Mogelvang et al. made attempts, indicating that lower variability is observed for the estimation of left ventricular mass than its volume. At the same time, these studies emphasized that the boundaries of consistency between different methods of estimation are too broad to accept clinically interchangeable use of different evaluation methods^10^. In their studies the main goal of which was to establish normative values for MRI of ventricular volume and mass in children, Buechel et al. showed that the variability of measurements between different researchers reaches 7% in the case of the left ventricle and 10% in the case of the right ventricle^11^. Also, Blalock et al. in a study dedicated to the assessment of the variability of the parameters of ventricular dimensions and function in MRI, indicated a greater variability in the measurements of the right ventricle, especially in the measurements of the right ventricular mass made based on images obtained in diastole^12^.

Optimistic data on the significance of the observed variability in the measurements of left ventricular volume, mass and ejection fraction was provided by the study conducted by Moody et al., which documented that short-term measurement errors between examinations do not differ significantly from long-term changes observed over a year. Thus, it was shown that the variability of left ventricle volume and mass measurements from MRI, resulting from technical factors, remains minimal, and even slight temporal changes in these parameters can be attributed to pathogens^13^. Interesting data on the discussed issue was provided by the publication of Karamitsos et al., who attempted to compare the repeatability of left ventricle mass and function measurements between experienced and inexperienced researchers, as well as to assess the impact on the discussed repeatability of brief intensive training of inexperienced researchers. This type of training improved the variability of left ventricular ejection fraction assessment from 7.2 to 3.7% and left ventricular mass from 7.7 to 6.7%^14^. In the light of the above-mentioned studies, the variability of LVEDD measurement obtained in our study, is a variability which may be considered as possible to omit, but at the same time indicates the optimal method of measurement.

An attempt to optimize the method of LVEDD measuring in the context of the accuracy of LVEDV prediction in the existing literature, according to the authors’ knowledge, has not been undertaken.

At the same time, it should be noted that the above gap in the studies conducted so far may result from a significantly lower “popularity” of LVEDD as a parameter used in research with the use of CMR than, for example, in research where the method is based on echocardiography. In recent years, only a few published studies have paid more attention to the importance of LVEDD measuring in CMR. An important study of this type was the study conducted by Neisius et al., comparing the ability of MRI and echocardiography to predict left ventricular remodelling after aortic valve replacement. Linear measurement of left ventricular dimensions in echocardiography is used in assessing the severity of aortic valve disease. These studies show that echocardiography understates the LVEDD measurement by an average of 6.6 mm. Moreover, the correlation of left ventricular remodelling after aortic valve replacement with the severity of the aortic valve defect estimated by CMR was stronger than in the case of echocardiography^15^. It is worth mentioning that, as documented in the study by Polte et al., the underestimation of echocardiographic measurements of left ventricular dimensions increases successively from linear, through superficial, to volumetric measurements^16^. Disha et al. also noted that changes in the morphological and functional parameters of the left ventricle after aortic valve replacement have different dynamics depending on the native type of aortic valve: tricuspid and mitral^17^. The importance of LVEDD measuring in MRI may also be demonstrated by studies on the pathogenesis of myocardial infarction. Li et al. compared the predictive accuracy of CMR and echocardiography in interventional therapy in patients with acute myocardial infarction, showing that MRI parameters (LVEDD, LVESD and LVEF) are characterized by higher sensitivity and accuracy than the corresponding echocardiographic parameters^18^. Neuschl et al. analysed the correlation between left ventricular morphology and function in patients with severe post-infarction systolic dysfunction of the left ventricle with the presence of malignant arrhythmias. It has been documented that among the parameters of CMR, a lower LVEDD and a lower LVESD constitute risk factors for more frequent malignant arrhythmias in this group of patients^19^. In recent years, LVEDD has also been considered as one of the important parameters in studies on the determination of normative values for the assessment of the left ventricle by CMR. Macedo et al. stated that the normative values of measurements used in CMR mainly originate from studies conducted in North American and European populations. Therefore, they estimated such norms in the healthy Latin American population, showing values of 48 ± 5 mm for LVEDD^20^.

The authors see several significant limitations in the currently discussed study. In terms of the study group, the relatively small number of patients included in the project, the overrepresentation of men in the study group and the heterogeneity of the group resulting from the presence of various disease entities and conditions in the study group should be considered as such. On the other hand, the methodological shortcomings of the study include the short period of time from which the results were analysed; use of LVEDV instead of LVEDV/BSA as the parameter based on which left ventricular enlargement, and the assumption of limiting the study to end-diastolic measurement instead of verifying the suitability of both end-diastolic and end-systolic measurements. The above limitations do not affect the usefulness of the conducted study but may constitute grounds for further research.

## Conclusions

The measurement made in the short axis should be considered the optimal method to LVEDD measure in CMR, considering the repeatability of measurements and the accuracy of left ventricular enlargement prediction.

## Data Availability

Study data can be made available upon documented request.
